# Supplementary material for: Validation of Novel Molecular Imaging Targets Identified by Functional Genomic mRNA Profiling to Detect Dysplasia in Barrett’s Esophagus
Source: Cancers (Basel). 2022 May 17;14(10):2462. doi: 10.3390/cancers14102462 (PMC9139936; doi:10.3390/cancers14102462)
Supplement: Supplementary file 1 [file cancers-14-02462-s001.zip › cancers-1693895-supplementary.pdf]

Validation of Novel Molecular Imaging Targets Identified by Functional Genomic mRNA Profiling to Detect Dysplasia in Barrett´s Esophagus

Journal: *Cancers*

Supplementary materials

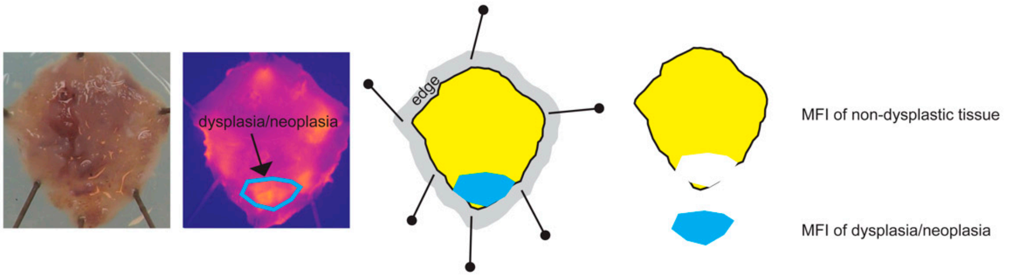

Figure S1 The diagram for the calculation of mean fluorescence intensity correlated to histology on fresh specimens. The blue circle highlighted the dysplastic or neoplastic tissue after histology correlation of bread-loaf slices. The yellow color highlighted the non-dysplastic tissue. Mean fluorescence intensity (MFI) of different histology type tissue was then calculated in Image J according to the delineations. MFI: mean fluorescence intensity.

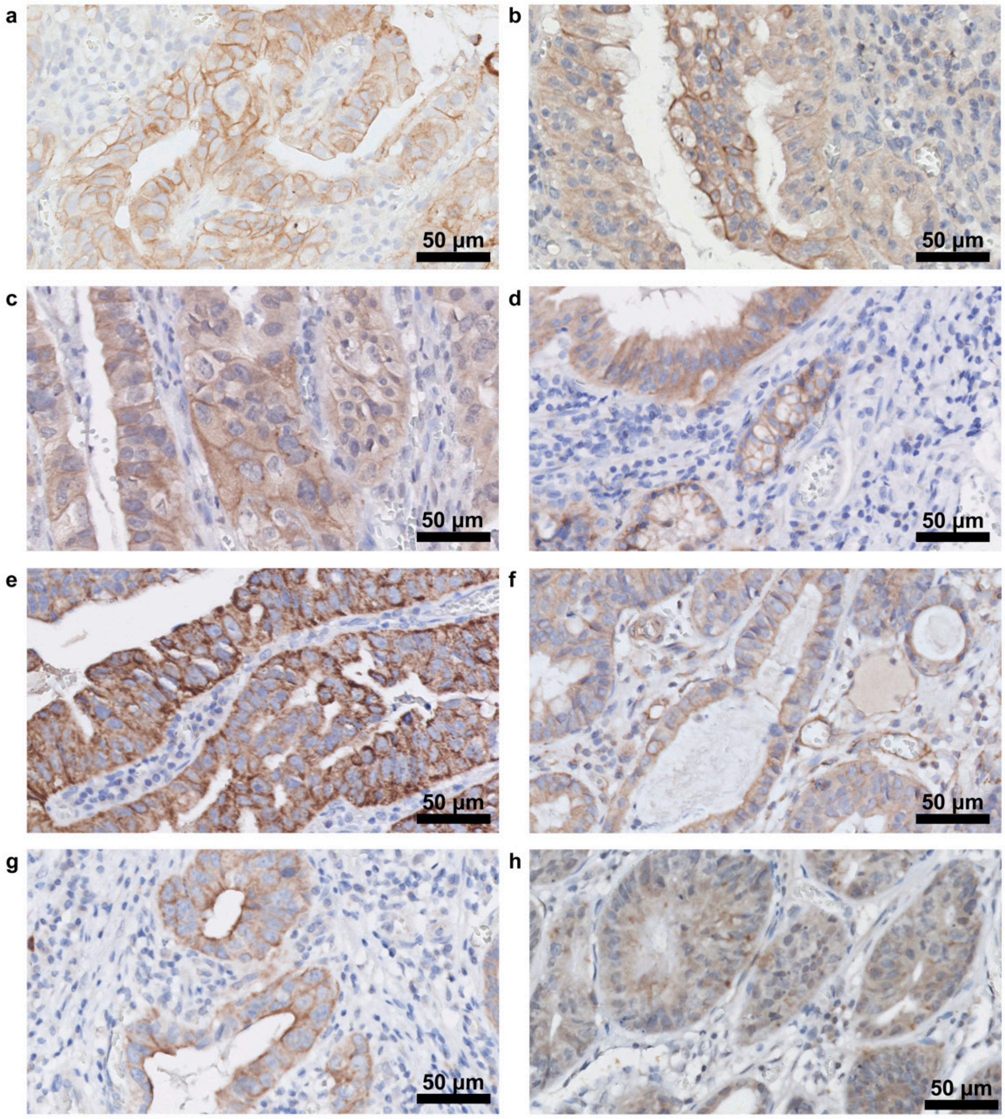

Figure S2 The subcellular localization of the proteins. CDH11 (**a**) shows plasma membranous staining; ERBB3 (**b**) shows mainly cell membranous, sometimes cytoplasmic staining; TMPRSS4 (**c**), DDR1 (**d**), PKCι (**e**), SPARC (**f**) and SULF1 (**g**) show mainly cytoplasmic, sometimes membranous staining; GREM1 (**h**) shows cytoplasmic staining.

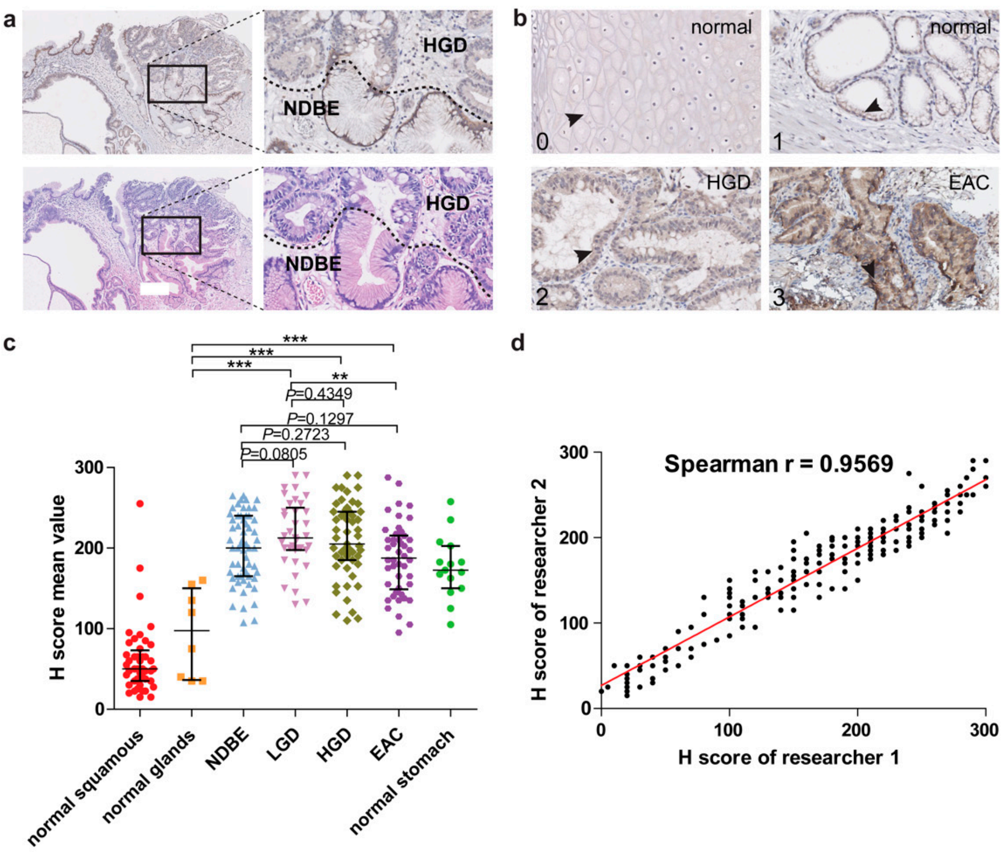

Figure S3 Immunohistochemistry results of TMPRSS4. (**a**) The IHC images in low and high magnification, accompanied by H&E staining results in serial cutting slices and pathological delineation. (**b**) It explains how we define staining intensity of 0 to 3. (**c**) The comparison of mean H score among seven groups, including normal squamous epithelium, normal glands, NDBE, LGD, HGD, EAC, normal stomach. Two researchers performed H scoring separately and blindly. The H score mean value means the average value of H score of researcher 1 and researcher 2. (**d**) The correlation analysis of H scoring by researcher 1 and researcher 2, with Spearman correlation. ** *P* value < 0.001; *** *P* value < 0.0001. EAC: esophageal adenocarcinoma; HGD: high grade dysplasia; LGD: low grade dysplasia; NDBE: non-dysplastic Barrett's esophagus.

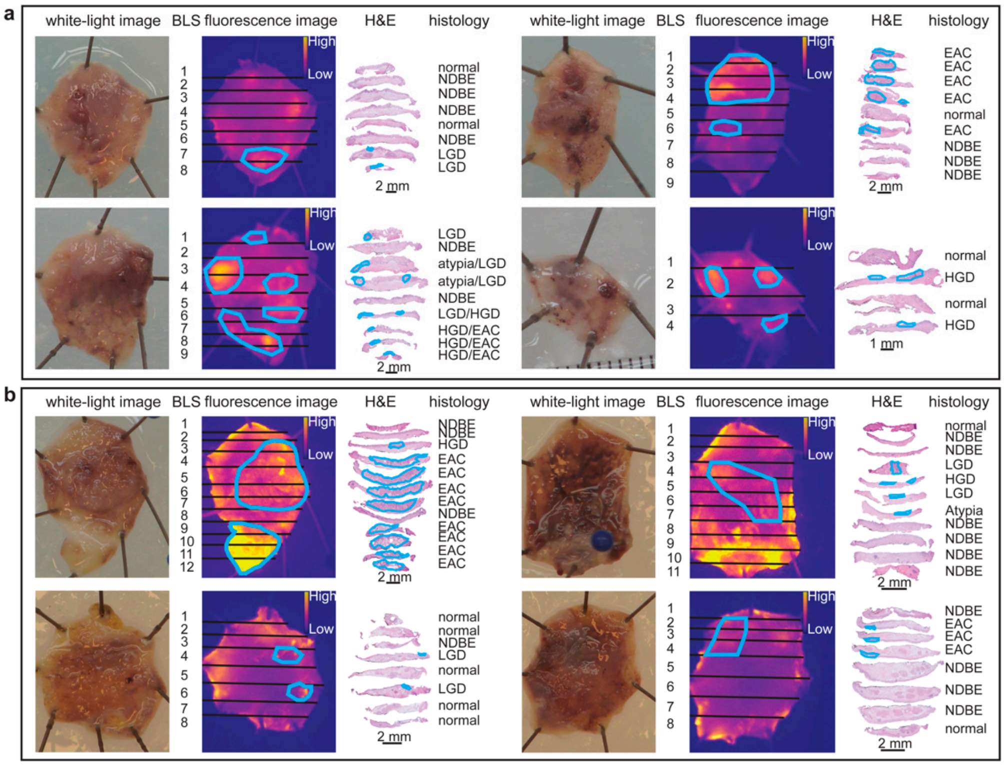

Figure S4 The histology correlation overview of SPARC-800CW and SULF1-800CW

(**a**) The fluorescence of SPARC-800CW after incubation and rinsing on endoscopic mucosal resection (EMR) specimens. (**b**) The fluorescence of SULF1-800CW after incubation and rinsing on EMR specimens. In the left is the white light image; in the middle is the fluorescence image; in the right are the H&E images of bread-loaf slices. The dysplastic BE tissue was delineated with blue circled lines. Due to the coagulation effect caused during in-vivo EMR procedures, the edge of the specimens showed high fluorescence. BLS: bread-loaf slices; EAC: esophageal adenocarcinoma; HGD: high grade dysplasia; LGD: low grade dysplasia; NDBE: non-dysplastic Barrett's esophagus; normal: normal tissue including normal squamous epithelium and normal stroma tissue.

Table S1. The characteristics of 25 patients included for immunohistochemical staining (in excel format)

Table S2. The utilized primary antibodies and optimized immunohistochemistry protocols of nine candidate genes.

Primary antigen	Mono /poly-clonal	Company	Concentration /Volume	Dilution	Antigen-retrieval
**#9 GREM1**	Polyclonal	invitrogen	1mg/ml	1:250	Tris/HCL
**#11 SPARC**	Monoclonal	ThermoFisher	2 mg/ml	1:1000	Tris/EDTA
**#12 ENPEP**	Monoclonal	abcam	100ul	1:50	Tris/EDTA & Tris/HCL
**#15 SULF1**	Polyclonal	invitrogen	100ul	1:100	Tris/HCL
**#24 ERBB3**	Monoclonal	OriGene	1mg/ml	1:50	EDTA
**#26 PKCι**	Monoclonal	Santa Cruz	200ug/ml	1:250	Tris/HCL
**#50 CDH11**	Monoclonal	ThermoFisher	500ug/ml	1:100	Citrate (pH6.0)
**#72 DDR1**	Monoclonal	USBiological	100ul	1:500	EDTA
**#111 TMPRSS4**	Polyclonal	Proteintech	150ul	1:1000	Tris/EDTA

Table S3. The overview of all overexpressed genes in esophageal adenocarcinoma compared to Barrett's esophagus by FGmRNA profiling (in excel format).

Table S4. Top 500 overexpressing genes of FGmRNA profiling in esophageal adenocarcinoma compared to Barrett's esophagus.

**1**	MGP	**51**	CD52	**101**	MME	**151**	ATP11A
**2**	IGF2BP3	**52**	THEM6	**102**	RASSF7	**152**	IDS
**3**	ADAP1	**53**	MAPRE1	**103**	NSFL1C	**153**	PPIA
**4**	ICA1	**54**	MS4A6A	**104**	TMEM106B	**154**	ELMO3
**5**	ACOT8	**55**	STAU1	**105**	F8A1,F8A2,F8A3	**155**	ARFGAP1
**6**	IGFBP7	**56**	H2AFV	**106**	HSPD1	**156**	OSER1
**7**	GPR137B	**57**	BARX1	**107**	SMCHD1	**157**	TUFT1
**8**	TYROBP	**58**	SDC4	**108**	LAPTM5	**158**	CHMP3
**9**	GREM1	**59**	LTK	**109**	STAT6	**159**	AHCY
**10**	UBE2C	**60**	WIPI2	**110**	COBL	**160**	TMEM189
**11**	SPARC	**61**	CYP2B6	**111**	TMPRSS4	**161**	SCAMP3
**12**	ENPEP	**62**	SKAP2	**112**	BLCAP	**162**	SMG5
**13**	YWHAB	**63**	CD14	**113**	KLF12	**163**	DDX52
**14**	LTBP2	**64**	RAMP1	**114**	SLMO2	**164**	DDX27, SS18
**15**	SULF1	**65**	GPSM2	**115**	IFT52	**165**	ZNF12
**16**	GAST	**66**	MYBL2	**116**	EFEMP2	**166**	CEBPB
**17**	FSTL1	**67**	XPO1	**117**	ZMYM1	**167**	TNFRSF25
**18**	LAMA4	**68**	SNRPB	**118**	FRZB	**168**	MRPS18A
**19**	RAB40B	**69**	CD248	**119**	PRH1-PRR4	**169**	GATAD1
**20**	MTMR11	**70**	TCF7L2	**120**	CLIP2	**170**	C1R
**21**	EIF3B	**71**	MEG3	**121**	HDHD3	**171**	LBH
**22**	HS2ST1	**72**	DDR1	**122**	LAMB3	**172**	RALGAPB

**23**	FOXA2	**73**	AGT	**123**	TNFAIP6	**173**	LLGL2
**24**	ERBB3	**74**	CTSA	**124**	C6orf123	**174**	DPH3P1, TCFL5
**25**	COL5A2	**75**	VEGFA	**125**	LONRF3	**175**	EPS8L3
**26**	PRKCI	**76**	MTMR1	**126**	OSBPL3	**176**	PQBP1
**27**	DDAH2	**77**	AMIGO2	**127**	CDK8	**177**	WWP1
**28**	TNNC2	**78**	APOD	**128**	TTC22	**178**	NID2
**29**	DKK3	**79**	OGN	**129**	SPATA7	**179**	PSMB2
**30**	GLS	**80**	SORD	**130**	TRAC	**180**	CD2
**31**	FZD5	**81**	DPM1	**131**	HMGB1	**181**	TGIF2-C20orf24
**32**	CXCL13	**82**	CTSK	**132**	TMEM176A	**182**	SEPT7
**33**	COL9A1	**83**	ARHGAP12	**133**	PLEKHB1	**183**	PDS5B
**34**	BZW2	**84**	JUP	**134**	C1orf106	**184**	APOOL
**35**	ADD3	**85**	SERINC3	**135**	DDX27	**185**	VBP1
**36**	SMAD6	**86**	TAX1BP1	**136**	ADNP	**186**	PLXDC1
**37**	TTC30A	**87**	FKBP1A	**137**	AGR2	**187**	RAB25
**38**	PSMB4	**88**	ESD	**138**	MRPL13	**188**	SDS
**39**	C1orf27	**89**	SIGIRR	**139**	FNBP1L	**189**	TPP2
**40**	ELF3	**90**	EPN3	**140**	EPB41L1	**190**	PPIA
**41**	GFRA1	**91**	KCNK1	**141**	F2R	**191**	BGN
**42**	ATP9A	**92**	ACVR1B	**142**	DIRAS3	**192**	PTPRC
**43**	ZGPAT	**93**	OGT	**143**	BCAP31	**193**	EDN3
**44**	GPX2	**94**	ZNF239	**144**	RSU1	**194**	RPE65
**45**	PELI2	**95**	PSMA1	**145**	GPM6A	**195**	PLEKHA6
**46**	HLA-DPA1	**96**	ENOX2	**146**	PIK3C2B	**196**	VAPA
**47**	LSR	**97**	MTCH1	**147**	SOX9	**197**	COL4A1
**48**	COL1A2	**98**	RAD54B	**148**	KIF2C	**198**	OPCML
**49**	MMP1	**99**	FANCF	**149**	PROCR	**199**	DNAJB2
**50**	CDH11	**100**	HEATR1	**150**	CUL4A	**200**	SAP18
**201**	PSMA7	**251**	DDX47	**301**	SLC2A1	**351**	COA1
**202**	TAGLN2	**252**	ZNF75D	**302**	PSEN2	**352**	ANGPT1
**203**	PRKCA	**253**	ITGBL1	**303**	CTTN	**353**	STX16
**204**	CREBZF	**254**	ZAK	**304**	HSPE1	**354**	POFUT1
**205**	C1orf116	**255**	TGM2	**305**	LRRC32	**355**	POP1
**206**	MS4A4A	**256**	PRSS8	**306**	RPIA	**356**	HPRT1
**207**	TMEM185B	**257**	RAB22A	**307**	ERGIC3	**357**	GRB10
**208**	ITGA4	**258**	MXRA5	**308**	RNF128	**358**	NCALD
**209**	SH3BGRL	**259**	SLAMF1	**309**	ARFGEF2	**359**	WIPF2

**210**	DPY19L4	**260**	SP140L	**310**	FYB	**360**	ALG3		
**211**	IL17RB	**261**	ZKSCAN1	**311**	FEZ1	**361**	POLD2		
**212**	RCBTB1	**262**	SLC12A9	**312**	TRAPPC6A	**362**	ASPN		
**213**	PCBP2	**263**	MAPK14	**313**	COL3A1	**363**	GGCX		
**214**	ZMYM2	**264**	EIF2AK1	**314**	MOSPD2	**364**	SMAGP		
**215**	TNFSF10	**265**	TMCO1	**315**	AAK1	**365**	ERBB2		
**216**	ZNF32	**266**	AC104699.1	**316**	TPX2	**366**	AURKAPS1, RAB3GAP2		
**217**	PCMTD2	**267**	MEST	**317**	PKP3	**367**	ARRB1		
**218**	INTS1	**268**	OTTHUMG00000003319	**318**	NF1	**368**	HLA-DRB1, HLA-DRB3, HLA-DRB4		
**219**	IKBKE	**269**	CBLC	**319**	BORA	**369**	RPL37A		
**220**	PRPF6	**270**	ENTPD1	**320**	GABBR1, UBD	**370**	CYP4A11		
**221**	SERPINA4	**271**	LZTS3	**321**	ITPKA	**371**	CYP2B6		
**222**	IFITM3	**272**	HLA-DMB	**322**	ATR	**372**	CR1		
**223**	DYNLRB1	**273**	RBP4	**323**	TAF2	**373**	PLEKHG6		
**224**	UGGT2	**274**	YME1L1	**324**	TSPAN8	**374**	GORASP2		
**225**	YTHDF1	**275**	TPP1	**325**	YWHAZ	**375**	VIL1		
**226**	FOXH1	**276**	CLEC4A	**326**	LSM5	**376**	HOXA1		
**227**	PSMA2	**277**	TNFSF11	**327**	GULP1	**377**	XPNPEP2		
**228**	CTBP2	**278**	DIS3	**328**	CMPK1	**378**	ARHGEF4		
**229**	CHCHD2	**279**	COL1A1	**329**	TP53TG1	**379**	RERGL		
**230**	IL13RA1	**280**	KPNA3	**330**	GGCT	**380**	CCZ1, CCZ1B		
**231**	RALYL	**281**	LCN2	**331**	MORC4	**381**	SCAMP5		
**232**	SSR3	**282**	SNX13	**332**	SPON2	**382**	GAD1		
**233**	NFKBIE	**283**	AURKA	**333**	AAR2	**383**	SLC9A8		
**234**	PLAGL2	**284**	FAM49A	**334**	ISG20L2	**384**	NEK3		
**235**	OVOL2	**285**	SPR	**335**	SH3GLB1	**385**	AP5Z1, MIR4656		
**236**	DLGAP4	**286**	CXorf57	**336**	UBE2V1P9	**386**	TGIF2		
**237**	ADIPOQ	**287**	GYPA	**337**	C3	**387**	THY1		
**238**	KPNA4	**288**	EMD	**338**	TRAC	**388**	DBI		
**239**	PGAP3	**289**	TMEM176B	**339**	PLAUR	**389**	DROSHA		
**240**	GDNF	**290**	UFC1	**340**	RAD21	**390**	VCAM1		
**241**	RAB31	**291**	RASSF2	**341**	TXNDC9	**391**	HSPA14		
**242**	U2SURP	**292**	HDAC1	**342**	PPARGC1A	**392**	NCOA1		
**243**	TTC17	**293**	PLOD3	**343**	SPSB1	**393**	NPL		
**244**	PRSS2, PRSS3	**294**	HEBP2	**344**	MUC1	**394**	ZFP36L2		
**245**	CR2	**295**	KDELR2	**345**	ZNF217	**395**	S100P		

**246**	HNF1A	**296**	NQO1	**346**	AVL9	**396**	TBC1D4
**247**	AKAP11	**297**	CCT6A	**347**	ESM1	**397**	FOLH1, FOLH1B
**248**	AKR1C3	**298**	COMMD3-BMI1	**348**	PSMF1	**398**	ORC2
**249**	NR4A3	**299**	BAD	**349**	ABCB6	**399**	STAG2
**250**	MFAP5	**300**	PLA2G7	**350**	PRPS1	**400**	FGF12

**401**	RAB17	**426**	GTF3A	**451**	PTPRR	**476**	PDK2
**402**	GUCY1B3	**427**	LGALS8	**452**	MTERFD1	**477**	GATA4
**403**	PEX3	**428**	DICER1	**453**	RARA	**478**	YKT6
**404**	SLC5A5	**429**	TFEB	**454**	UBE2D4	**479**	CSNK2A1
**405**	ATP6V1C1	**430**	DNASE1	**455**	RAC1	**480**	C11orf49
**406**	GLI1	**431**	CSE1L	**456**	NDRG3	**481**	MICU2
**407**	FAM50A	**432**	DNPH1	**457**	EIF3F	**482**	MYOC
**408**	SSB	**433**	STIL	**458**	KCNMB3	**483**	PRR15L
**409**	FTSJ2	**434**	TRIM44	**459**	EFNA1	**484**	NCOA6
**410**	ZNF318	**435**	HLA-DPB1	**460**	PDZK1IP1	**485**	CST4
**411**	IGSF6	**436**	LAD1	**461**	AGO1	**486**	ZC3H3
**412**	PGK1	**437**	RPL39	**462**	PRRC2C	**487**	MCF2L-AS1
**413**	POR	**438**	CUX1	**463**	CKAP5	**488**	AKAP4
**414**	ANKEF1	**439**	RPS3	**464**	ARHGEF7	**489**	TCF4
**415**	SCNM1, TNFAIP8L2-SCNM1	**440**	EEF1D	**465**	EFR3A	**490**	FAR2
**416**	ESRP1	**441**	MYRF	**466**	UCKL1	**491**	WNT2
**417**	NELFCD	**442**	CFLAR	**467**	PPIH	**492**	SUN1
**418**	BAIAP2L2	**443**	IFITM2	**468**	CACYBP	**493**	KRT18
**419**	GCLC	**444**	HSP90AB1	**469**	NRP1	**494**	STK39
**420**	ATXN7L3B	**445**	FERMT1	**470**	AMMECR1	**495**	EIF6
**421**	PDE6D	**446**	TMPRSS2	**471**	MOSPD1	**496**	PYGM
**422**	HLA-DQB1	**447**	XPOT	**472**	INTS8	**497**	CNPY3
**423**	MCAM	**448**	KCNJ8	**473**	CHRDL1	**498**	LGALS4
**424**	HMHA1	**449**	SNRNP200	**474**	WNT4	**499**	TOX3
**425**	NUDT15	**450**	TTK	**475**	CD1E	**500**	HIST1H2BD

Table S5. Database search overview of the top 150 overexpressing genes by FGmRNA profiling.

RANK	GENE	Location	Location (GeneCards)	Location (ICAN)	IHC esophagus	IHC GE	Carcinogenesis	Imaging
8	TYROBP	M	M	Cell surface, IC	None	None	Shabo I, et al. [33]	None
9	GREM1	S	EC	Cell surface	Laurila, et al. [34]		Karagiannis GS, et al. [35]	None
11	SPARC	IC,S	EC, N	Basement M, platelet alpha granule lumen, cell surface	Yamashita K, et al. [36] Miura JT, et al. [37]	Lussier C, et al. [38]	Nagaraju GP, et al. [39]	None
12	ENPEP	M	M, EC, lysosome	Apical part of cell, apical M, brush border	None	Yuan Y, et al. [40]	Aravalli RN, et al. [41]	None
15	SULF1	IC,S	M, ER, EC, golgi apparatus	Cell surface, M raft, Golgistack	None	Gopal G, et al. [42]	Lai JP, et al. [43]	Pospisil P, et al. [44]
24	ERBB3	IC,M,S	EC,M	M	Yoon HH, et al. [45] Paterson AL, et al. [46] Nishigaki H, et al. [47]	None	Mang article, genecards.org gives a good summary	Terwisschavan Scheltinga AG, et al. [48]
26	PRKCI	IC	Cytosol, EC, N, cytoskeleton	Microtubule, cytoskeleton protein complex, cell leading edge	None	Yang YL, et al. [49]	Yang YL, et al. [50]	None
31	FZD5	M	M	Bicellular tight junction, Golgi M, cell surface	None	None	Snow GE, et al. [51]	None
41	GFRA1	M	M, EC	Anchored component of M, extrinsic component of M	None	None	Liu Z, et al. [52]	None
46	HLA-DPA1	IC,M,S	ER, endosome, lysosome, M, golgi apparatus	ER to Golgi transport, vesicle membrane, cell surface	None	None	None	None
50	CDH11	IC, M, S	M, EC	Not assigned	None	Torres S, et al. [53]	Yao J, et al. [54]	None

51	CD52	M	M, EC	Anchored component of M	None	None	Involved in the leukemia's	None
54	MS4A6A	IC,M	M	Not assigned	None	None	None	None
58	SDC4	M	EC,M, golgi apparatus	Focal adhesion, cell surface	None	None	None	None
59	LTK	M,S	M	Not assigned	None	None	Roll JD, et al. [55]	None
63	CD14	S	M, EC, golgi apparatus, endosome	external side of membrane, receptor complex, membrane microdomain, anchored component of membrane, endosomal part, endocytic vesicle	It is expressed on monocytes. [56]		Companioni O, et al. [57]	None
64	RAMP1	M	M	Transporter complex	None	None	Logan M, et al. [58]	None
69	CD248	M	EC, M	proteinaceous extracellular matrix, collagen trimer	None	TEM1 is expressed in several types of mesenchymal cells. [59]	None	Cicone F, et al. [60]
72	DDR1	IC, M	M	Basolateral plasma membrane, apicolateral plasma membrane	Sugimoto K, et al. [61] Nemoto T, et al. [62]	Hur H, et al. [63]	Jin H, et al. [64]	None
77	AMIGO2	M	M, N	Not assigned	None	None	None	None
83	ARHGAP12	IC	M, cytosol	Side of membrane	None	None	None	None

84	JUP	IC, M, cell junctions	M, N, EC, cytoskeleton, cytosol	Desmosome, apicolateral plasma membrane, intermediate filament cytoskeleton, lateral plasma membrane, cell cell junction, intercalated disc	It shows down regulated expression in ESCC compared to normal esophagus. [65]		Fredericks E, et al. [66]	None
85	SERINC3	M	M	Autophagosome	None	None	Bossolasco M, et al. [67]	None
86	TAX1BP1	IC, M	cytosol	plasma membrane protein complex	None	None	None	None
87	FKBP1A	IC, M	M, ER, cytosol	chromosomal region	None	None	None	None
89	SIGIRR	IC, M	M	Not assigned	None	None	None	None
91	KCNK1	M	M	Apical part of cell, apical plasma membrane, plasma membrane protein complex, cluster of actin based cell projection, cell projection membrane, brush border, plasma membrane receptor complex, lateral plasma membrane	None	None	None	None
96	ENOX2	IC, M	M, EC, cytosol	Side of membrane, external side of plasma membrane, secretory vesicle	None	None	None	None

101	MME	IC, M	M, endosome, golgi apparatus	Not assigned	Lee KW, et al. [68]	None	Lee KW, et al. [68] reported that MME can facilitate tumorigenesis; HoweverCheng CY, et al. [69] showed controversial results.	None
108	LAPTM5	Cytosol, M	M, lysosome	MHC protein complex	None	None	Nuylan M, et al. [70]	None
111	TMPRSS4	IC, M	M	Apical plasma membrane	None	Huang A, et al. [71, 72]	Huang A, et al. [71]	Cohen AS, et al. [73]
132	TMEM176A	M, mitochondria	Not assigned	Plasma membrane protein complex	Down regulated expression in ESCC compared to adjacent normal esophagus tissue. [74]		None	None
140	EPB41L1	IC, M	Cytoskeleton, cytosol, M	Apical part of cell	None	None	None	None

141	F2R	IC, M	M, EC, endosome, golgi apparatus	Plasma membrane raft, neuromuscular junction, cell substrate junction	None	None	None	None
143	BCAP31	M, ER	M, ER, cytosol	Side of membrane, endoplasmic reticulum golgi intermediate compartment membrane, etc.	None	BCAP31 is localized in cytoplasm and ER membrane. [75]	Wang A, et al. [76]	None
145	GPM6A	IC, M	M	Cell body, axon part, postsynapse, neuron spine, axon, dendrite, site of polarized growth, axonal growth cone, filopodium, postsynaptic membrane	None	None	None	None
149	PROCR	IC, M	M, EC	Cell substrate junction, anchoring junction, plasma membrane protein complex	None	None	Wang Q, et al. [77]	Dou WT, et al. [78]

The top 150 genes with protein products predicted to be localized on plasma membrane were listed in the table. Abbreviations: EC: extracellular; GE: gastroesophageal; IC: intracellular; IHC: immunohistochemistry; M: membrane; N: nucleus; S: secreted.

Table S6. The anti-TMPRSS4 immunohistochemistry results in dysplastic Barrett's esophagus and non-dysplastic Barrett's esophagus.

**Targets**	**H-score DBE**			**High (201-300)**		**Intermediate (101-200)**		**Negative-low (0-100)**	
	n	Mean (SD)	Mean value	n	%	n	%	n	%
**TMPRSS4**	138	204 (46)	High	71	51.45	66	47.83	1	0.72
	**H-score NDBE**			**High (201-300)**		**Intermediate (101-200)**		**Negative-low (0-100)**	
	n	Mean (SD)	Mean value	n	%	n	%	n	%
**TMPRSS4**	59	200 (43)	Intermediate	29	49.15	30	50.85	0	0

Table demonstrating IHC results of five targets in DBE and NDBE. Mean intensities and standard deviation within different categories are shown using the H-score. DBE: dysplastic Barrett's esophagus; NDBE: non-dysplastic Barrett's esophagus; SD: standard deviation.
